# Requirement of IFT-B–BBSome complex interaction in export of GPR161 from cilia

**DOI:** 10.1242/bio.043786

**Published:** 2019-08-30

**Authors:** Shohei Nozaki, Roiner Francisco Castro Araya, Yohei Katoh, Kazuhisa Nakayama

**Affiliations:** Department of Physiological Chemistry, Graduate School of Pharmaceutical Sciences, Kyoto University, Sakyo-ku, Kyoto 606-8501, Japan

**Keywords:** BBSome, Cilia, GPR161, IFT-B complex, Smoothened

## Abstract

The intraflagellar transport (IFT) machinery, which includes the IFT-A and IFT-B complexes, mediates bidirectional trafficking of ciliary proteins. In addition to these complexes, the BBSome, which is composed of eight subunits that are encoded by the causative genes of Bardet-Biedl syndrome (BBS), has been proposed to connect the IFT machinery to ciliary membrane proteins, such as G protein-coupled receptors, to mediate their export from cilia. However, little is known about the connection between the IFT machinery and the BBSome. Using the visible immunoprecipitation assay, we here identified the interaction between IFT38 from the IFT-B complex and BBS1, BBS2 and BBS9 from the BBSome. Furthermore, by analyzing phenotypes of *IFT38*-knockout cells exogenously expressing wild-type IFT38 or its mutant lacking the ability to interact with BBS1+BBS2+BBS9, we showed that knockout cells expressing the IFT38 mutant have restored ciliogenesis; however, similar to *BBS1*-knockout cells, they demonstrated significant accumulation of GPR161 within cilia upon stimulation of Hedgehog signaling. These results indicate that the IFT-B–BBSome interaction is required for the export of GPR161 across the ciliary gate.

## Abstract (precis)

**Summary:** We here determine an interface between the IFT-B complex and the BBSome involving IFT38 and BBS1+BBS2+BBS9, and show that the interaction is crucial for GPR161 export from cilia.

## INTRODUCTION

Cilia are organelles that project from the surfaces of various eukaryotic cells, and are supported by the axoneme, which is a microtubule-based scaffold. Cilia function as cellular antennae by mechanosensing extracellular stimuli, such as light and fluid flow, and chemosensing morphogenetic signals, such as Hedgehog (Hh) (Briscoe and Thérond, 2013; Mukhopadhyay and Rohatgi, 2014). Owing to their crucial roles, defects in cilia lead to a variety of congenital disorders, such as Bardet-Biedl syndrome (BBS), Joubert syndrome, nephronophthisis, Meckel syndrome, and short-rib thoracic dysplasia, which are collectively referred to as the ciliopathies, which accompany a wide spectrum of clinical manifestations, including retinal degeneration, polycystic kidneys, morbid obesity, and skeletal and brain malformations (Braun and Hildebrandt, 2017; Madhivanan and Aguilar, 2014). Although the ciliary membrane is continuous with the plasma membrane, the protein and lipid composition of the ciliary membrane differs greatly from that of the plasma membrane, due to the presence of the transition zone, which serves as a permeability/diffusion barrier at the base of cilia (Verhey and Yang, 2016; Wei et al., 2015).

In addition to structural components of the axonemal microtubules, such as the αβ-tubulin dimer, various soluble and membrane proteins, including G protein–coupled receptors (GPCRs), are specifically present within cilia and on the ciliary membrane. Therefore, ciliary assembly and the maintenance of ciliary functions strictly rely on the proper trafficking of these proteins, which is mediated by the intraflagellar transport (IFT) machinery, often referred to as IFT trains or IFT particles (Ishikawa and Marshall, 2011; Rosenbaum and Witman, 2002; Sung and Leroux, 2013). The IFT machinery contains the IFT-A and IFT-B complexes. It has been believed that the IFT-B complex mediates anterograde protein trafficking from the ciliary base to the tip powered by kinesin-2 motor proteins, whereas the IFT-A complex mediates retrograde trafficking with the aid of the dynein-2 complex (Ishikawa and Marshall, 2011; Nakayama and Katoh, 2018; Sung and Leroux, 2013; Taschner and Lorentzen, 2016). We as well as others recently demonstrated the overall architecture of the IFT-B complex, which is composed of 16 subunits (Boldt et al., 2016; Katoh et al., 2016; Taschner et al., 2016). The holocomplex can be divided into the core (IFT-B1) subcomplex composed of ten subunits and the peripheral (IFT-B2) subcomplex composed of six subunits; the two subcomplexes are connected by a tetrameric unit involving two core subunits, IFT52 and IFT88, and two peripheral subunits, IFT38/CLUAP1 and IFT57 (hereafter, the tetrameric unit is referred to as the ‘connecting tetramer’; see Fig. 2A for reference). Using the visible immunoprecipitation (VIP) assay, which we recently established to enable the convenient and flexible detection of protein–protein interactions (Katoh et al., 2018, 2015), we also demonstrated the architecture of the IFT-A complex, which is composed of six subunits, and associates with TULP3 (Hirano et al., 2017; Takahara et al., 2018); our model of the IFT-A architecture was compatible with those previously proposed for *Chlamydomonas* and mammalian IFT-A (Behal et al., 2012; Mukhopadhyay et al., 2010).

In addition to the IFT-A and IFT-B complexes, the BBSome moves along the axonemal microtubules in association with IFT particles at least in some cell types (Lechtreck et al., 2009; Williams et al., 2014), and participates in the removal of cargo membrane proteins from cilia by connecting them to the IFT machinery (Lechtreck et al., 2013, 2009; Liu and Lechtreck, 2018; Nachury, 2018; Ye et al., 2018). We previously clarified the overall architecture of the BBSome by the VIP assay, and demonstrated that it was composed of eight BBS proteins (see Fig. 2A for reference) (Katoh et al., 2015). In our BBSome model, which has been refined from the previously proposed models (Nachury et al., 2007; Zhang et al., 2012), four subunits, namely, BBS1, BBS2, BBS7 and BBS9, constitute the core subcomplex, with which the linker subcomplex, BBS4–BBS18–BBS8 associates via an interaction between BBS8 and BBS9. BBS5, which interacts with BBS9, probably mediates the association of the BBSome with the ciliary membrane via its pleckstrin-homology domain (Nachury et al., 2007). The BBSome architecture we determined is largely consistent with the model recently predicted from reconstitution of purified BBS proteins (Klink et al., 2017). In addition, the small GTPase ARL6/BBS3 regulates the membrane recruitment and coat-like assembly of the BBSome via an interaction with BBS1 (Jin et al., 2010; Liew et al., 2014; Zhang et al., 2011). We have recently shown that the ARL6–BBS1 interaction is reinforced by the binding of BBS9 to BBS1 (Nozaki et al., 2018).

In cells derived from knockout (KO) mice of IFT25 or IFT27/BBS19, retrograde trafficking of the BBSome and ciliary GPCRs, including Smoothened (SMO) and GPR161, both of which are components of Hh signaling, is severely impaired, although the assembly of cilia appears to be normal (Eguether et al., 2014; Keady et al., 2012; Mick et al., 2015); IFT25 and IFT27 form a heterodimer in the IFT-B core subcomplex (Bhogaraju et al., 2011; Nakayama and Katoh, 2018; Taschner and Lorentzen, 2016). As cells knocked out for a BBSome subunit and a regulator of the BBSome (ARL6/BBS3 or LZTFL1/BBS17) demonstrate similar defects in GPCR trafficking, the groups of Nachury and Pazour independently proposed that IFT25 and IFT27 regulate retrograde trafficking or ciliary export of these ciliary GPCRs mediated by the BBSome, although through distinct mechanisms by which IFT25–IFT27 functionally associate with the BBSome (Eguether et al., 2014; Liew et al., 2014). In addition, the BBSome was shown to regulate export of ciliary membrane proteins including GPCRs (Lechtreck et al., 2013, 2009; Liu and Lechtreck, 2018; Nozaki et al., 2018; Wingfield et al., 2018; Ye et al., 2018), and other studies showed that the BBSome interacts *in vitro* with peptides from intracellular regions of ciliary GPCRs, including SMO and GPR161 (Klink et al., 2017; Ye et al., 2018).

In this study, we addressed the possibility that the IFT machinery regulates BBSome function via a direct interaction. Using the VIP assay, we found that the IFT-B–BBSome interaction involves IFT38 from the IFT-B complex and BBS1, BBS2, and BBS9 from the BBSome. Furthermore, by analyzing phenotypes of *IFT38*-KO cell lines exogenously expressing an IFT38 deletion construct, we showed that the IFT-B–BBSome interaction is required for export from cilia of GPR161, a GPCR involved in Hh signaling.

## RESULTS

### IFT-B–BBSome interaction is mediated by IFT38 and BBS1–BBS2–BBS9

**Fig. 1.**
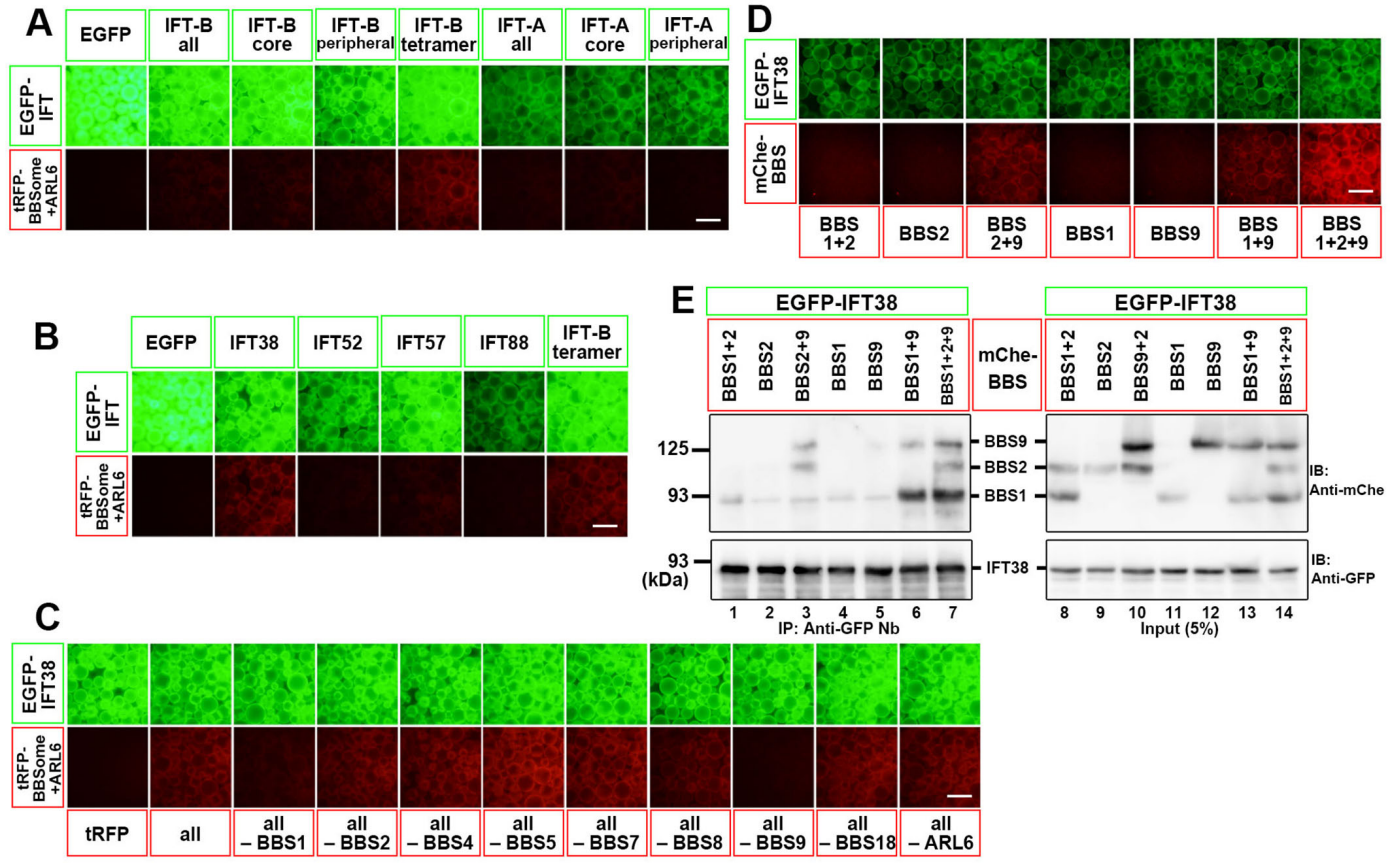
**Identification of an interaction between IFT38 and BBS1–BBS2–BBS9.** (A) Subunits of the IFT-B-connecting tetramer interact with the BBSome. HEK293T cells were co-transfected with expression vectors for EGFP-fused subunits of the IFT-B or IFT-A complex as indicated, and all the BBSome subunits plus ARL6 fused to mChe. 24 h after transfection, lysates were prepared from the transfected cells and immunoprecipitated with GST-tagged anti-GFP Nb pre-bound to glutathione-Sepharose 4B beads and processed for the VIP assay. (B) Identification of IFT38 as an IFT-B subunit responsible for BBSome interaction. Lysates were prepared from HEK293T cells coexpressing EGFP-fused IFT-B subunits as indicated, and all the BBSome subunits plus ARL6 fused to mChe, and subjected to the VIP assay. (C) Subtractive VIP assay to identify candidate BBSome subunits interacting with IFT38. Lysates prepared from HEK293T cells coexpressing EGFP-IFT38 and all but one (as indicated) subunits of the BBSome plus ARL6 fused to mChe were processed for the VIP assay. (D,E) Identification of BBS1, BBS2 and BBS9 as BBSome subunits responsible for the interaction with IFT38. Lysates prepared from HEK293T cells expressing EGFP-IFT38 together with mChe-fused BBSome subunit(s) indicated were processed for the VIP assay (D) or conventional immunoblotting analysis (E) using an anti-mChe antibody (upper panels) or an anti-GFP antibody (lower panels). Scale bars: 200 μm.

To find a potential interface between the IFT machinery and the BBSome, we used the VIP assay, which is a convenient and flexible strategy to visually detect not only one-to-one protein interactions but also one-to-many and many-to-many protein interactions (Funabashi et al., 2017, 2018; Hamada et al., 2018; Katoh et al., 2018, 2015, 2016). When lysates were prepared from HEK293T cells coexpressing either all IFT-B subunits or all the subunits of the IFT-B core or peripheral subcomplex fused to EGFP and all BBSome subunits plus ARL6 fused to TagRFP (tRFP) were immunoprecipitated with GST-tagged anti-GFP nanobodies (Nb) pre-bound to glutathione-Sepharose beads, red signals on the precipitated beads were below the detection level (Fig. 1A, columns 2–4). However, when EGFP-fused components of the IFT-B-connecting tetramer, IFT38/IFT52/IFT57/IFT88, were coexpressed with all the BBSome subunits fused to tRFP, weak but distinct red signals were detectable on the precipitated beads (Fig. 1A, column 5). When each subunit of the IFT-B-connecting tetramer fused to EGFP was separately coexpressed with tRFP-fused BBSome subunits, red signals were detected only in the case of EGFP-IFT38 (Fig. 1B). These results indicate that IFT38 in the IFT-B complex is involved in the IFT-B–BBSome interaction. On the other hand, we could not detect red signals when all the IFT-A subunits or all the IFT-A core or peripheral subunits fused to EGFP were coexpressed with all the BBSome subunits fused to tRFP (Fig. 1A, columns 6–8). It is thus unlikely that the IFT-A complex makes a major contribution to BBSome binding, although ‘no signal detection’ in the VIP assay does not necessarily imply ‘no interaction’ as described in our previous studies (Katoh et al., 2015, 2016).

**Fig. 2.**
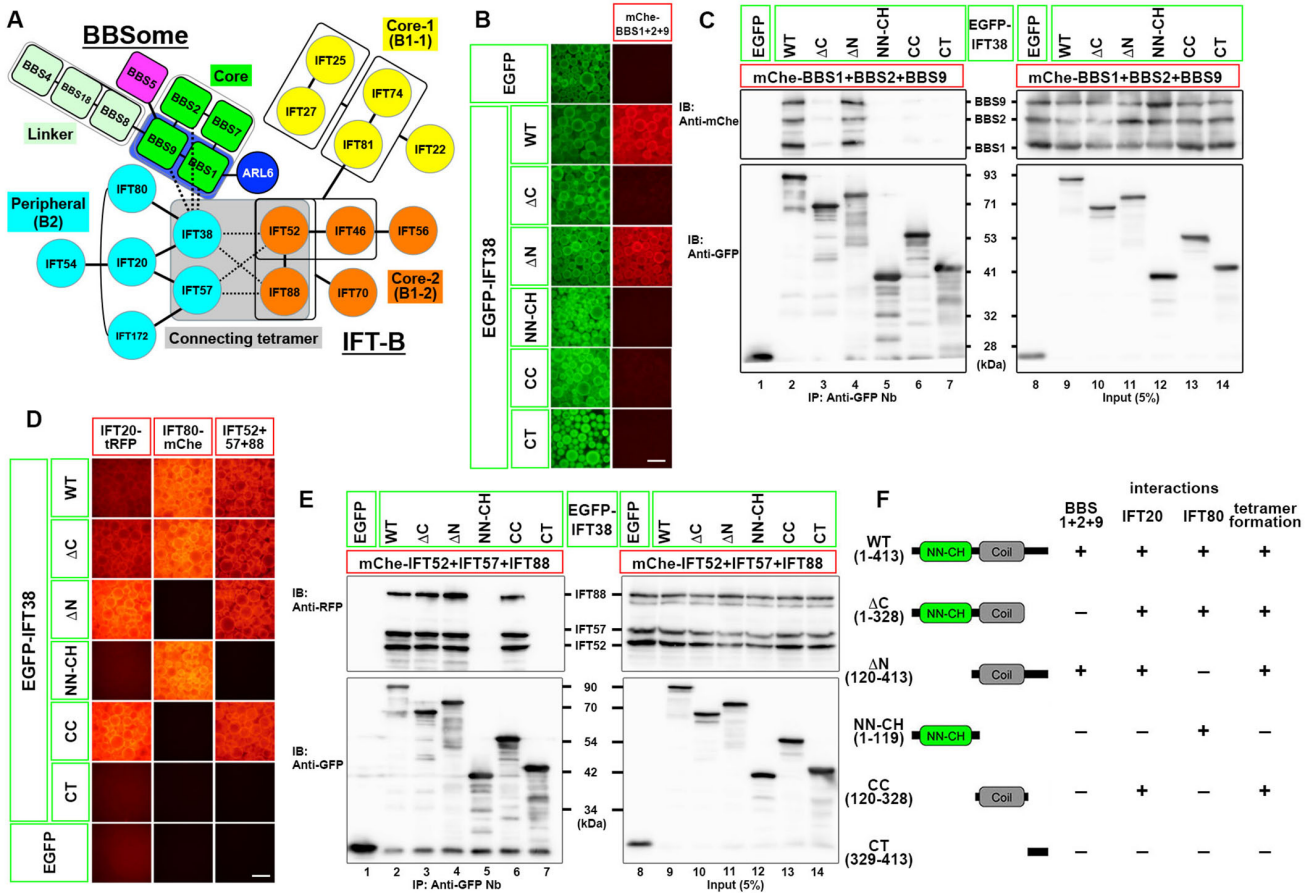
**Differentiation of the region of the IFT38 protein involved in BBSome binding from those involved in its interactions with other IFT-B subunits.** (A) A model for the interaction of the BBSome with the IFT-B complex predicted from the data shown in Fig. 1. (B,C) The CT region of IFT38 is essential for its interaction with the BBSome subunits. Lysates prepared from HEK293T cells expressing any of the EGFP-IFT38 constructs schematically shown in Fig. 2F (left side) together with mChe-fused BBS1+BBS2+BBS9 were processed for the VIP assay (B) or immunoblotting analysis (C) using an anti-mChe antibody (upper panels) or an anti-GFP antibody (lower panels). (D) Determination of regions of the IFT38 protein involved in its interactions with IFT20, IFT80, and IFT52+IFT57+IFT88 (the other subunits of the connecting tetramer). Lysates prepared from HEK293T cells expressing any of the EGFP-IFT38 constructs (Fig. 2F, left side) together with mChe-fused IFT20 (left column), IFT80 (middle column), or IFT52+IFT57+IFT88 (right column) were processed for the VIP assay. (E) Beads bearing EGFP-fused and mChe-fused IFT proteins used in the right column of Fig. 2E were processed for immunoblotting analysis using an anti-RFP antibody that reacts with mChe (upper panels) or an anti-GFP antibody (lower panels). (F) Summary of the results shown in Fig. 2B–E. (+), robust interaction; (−), no interaction. Scale bars: 200 μm.

We then addressed which BBSome subunit(s) participate in the IFT-B–BBSome interaction. To this end, we performed the subtractive VIP assay. When an individual subunit of the BBSome or ARL6 fused to tRFP was omitted from the VIP assay, red signals were diminished in the absence of tRFP-tagged BBS1 or BBS9 (Fig. 1C), suggesting potential involvement of these two BBSome subunits in the IFT-B–BBSome interaction. We then analyzed whether BBS1 and BBS9 indeed interact with IFT38. We also included BBS2 recently shown to directly interact with BBS9 (Katoh et al., 2015; Klink et al., 2017) and to co-immunoprecipitate with IFT38 (Beyer et al., 2018). No red signals were detected when mCherry (mChe)-fused BBS1, BBS2 or BBS9 alone was coexpressed with EGFP-IFT38 (Fig. 1D); in this experiment, we used mChe-fused BBS proteins instead of tRFP-fused ones, because the anti-tRFP antibody cross-reacts with EGFP, whereas neither of the anti-RFP antibody, which can detect mChe, and the anti-mChe antibody cross-reacts with EGFP. By contrast, red signals were detected when mChe-BBS1+BBS9 or mChe-BBS2+BBS9 were coexpressed with EGFP-IFT38, and robust red signals were detected when mChe-BBS1+BBS2+BBS9 were coexpressed (Fig. 1D). The VIP data were confirmed by conventional immunoblotting analysis (Fig. 1E); EGFP-IFT38 co-immunoprecipitated mChe-fused BBS1, BBS2, and BBS9 when they were coexpressed (lane 7). On the basis of these data, we conclude that the IFT-B component IFT38 makes a major contribution to the IFT-B–BBSome interface via interaction through BBS1, BBS2, and BBS9 from the BBSome (see Fig. 2A).

Although we could not detect interaction of the IFT-B core subunits with the BBSome subunits (Fig. 1A, row 3), previous studies implicated IFT25–IFT27 in the BBSome function (Eguether et al., 2014; Liew et al., 2014). We therefore examined whether IFT25–IFT27 directly interacts with the BBSome. As shown in Fig. S1A, however, we could not detect interaction of IFT25–IFT27 with all, core or linker subunits of the BBSome, or with BBS1+BBS2+BBS9. It had previously been suggested that IFT25–IFT27 disengaged from other IFT-B subunits interacts with the nucleotide-free form of ARL6 and promote ARL6 activation to drive BBSome assembly (Liew et al., 2014). However, as shown in Fig. S1B, our VIP assay failed to detect interaction of IFT25–IFT27 with ARL6(WT) and with ARL6(T31R), an ARL6 mutant that mimics the nucleotide-free and GDP-bound forms (Liew et al., 2014).

### C-terminal region of IFT38 is required for its interaction with BBS1, BBS2, and BBS9

We then set out to determine the region of IFT38 that is responsible for its interaction with the BBSome subunits, as we and others previously showed that IFT38 acts as a hub subunit in the IFT-B complex (Katoh et al., 2016; Taschner et al., 2016); it directly interacts with IFT20 and IFT80 in the peripheral subcomplex, and constitutes an interface between the core and peripheral subcomplex by forming the connecting tetramer together with IFT52+IFT57+IFT88 (Fig. 2A).

To this end, we utilized various IFT38 constructs, which were used in our previous study (Katoh et al., 2016). The IFT38 protein was predicted to have an N-terminal NDC80-NUF2 calponin homology (NN-CH) domain, followed by a coiled-coil (CC) region (Taschner et al., 2016). As shown in Fig. 2B, row four, an IFT38 construct (ΔN, residues 120–413) lacking the NN-CH domain retained the ability to interact with BBS1+BBS2+BBS9. By contrast, an IFT38 construct lacking the C-terminal (CT) region (ΔC, residues 1–328) did not interact with BBS1+BBS2+BBS9 (row three). On the other hand, both the CT construct (residues 329–413) and the CC construct (residues 120–328) on its own failed to interact with BBS1+BBS2+BBS9 (rows seven and six, respectively).

The VIP data were confirmed by conventional immunoblotting analysis. As shown in Fig. 2C, the IFT38(ΔN) construct co-immunoprecipitated BBS1+BBS2+BBS9 at a level comparable to IFT38(WT) (compare lane four with lane two). In striking contrast, the IFT38(ΔC) construct did not co-immunoprecipitate BBS1+BBS2+BBS9 (lane three). Furthermore, none of the other IFT38 deletion constructs that were analyzed co-immunoprecipitated BBS1+BBS2+BBS9 (lanes five to seven). Altogether, the CC and CT regions of IFT38 mainly participate in its interaction with the BBSome subunits.

The interaction mode of the IFT38 constructs with BBS1+BBS2+BBS9 can be distinguished from those with the other IFT-B subunits. As reported previously (Katoh et al., 2016), IFT38 directly interacts with IFT20 and IFT80 via its CC and NN-CH regions, respectively (Fig. 2D, columns one and two, respectively; also see Fig. 2F); the IFT38(ΔC) construct retained the ability to interact with both IFT20 and IFT80 (row two). On the other hand, IFT38 forms the connecting tetramer together with IFT52+IFT57+IFT88 to make an interface between the peripheral and core subcomplexes (see Fig. 2A). As shown in Fig. 2D, column three (also see Fig. 2F), the CC region of IFT38 participates in formation of the connecting tetramer; again, the IFT38(ΔC) construct retained the ability to form the connecting tetramer (row two). The ability of the IFT38(ΔC) construct to form the connecting tetramer was also confirmed by immunoblotting analysis. As shown in Fig. 2E, EGFP-IFT38(ΔC) co-immunoprecipitated mChe-fused IFT52, IFT57 and IFT88, to an extent comparable to that of EGFP-IFT38(WT) (compare lane three with lane two). By comparing the abilities of these IFT38 constructs to interact with the other IFT-B subunits summarized in Fig. 2F, we conclude that the IFT38(ΔC) construct has specifically lost the ability to interact with the BBSome subunits.

We also attempted to identify a BBS1, BBS2, or BBS9 mutant that specifically loses the ability to interact with IFT38 but retains the ability to interact with other BBSome subunits and ARL6. However, our attempts have been unsuccessful so far, as these BBSome subunits interact with various BBSome subunits and ARL6 (see Fig. 2A; also see Nozaki et al., 2018).

### IFT38(WT) and IFT38(ΔC) differentially restore ciliogenesis in IFT38-KO cells

**Fig. 3.**
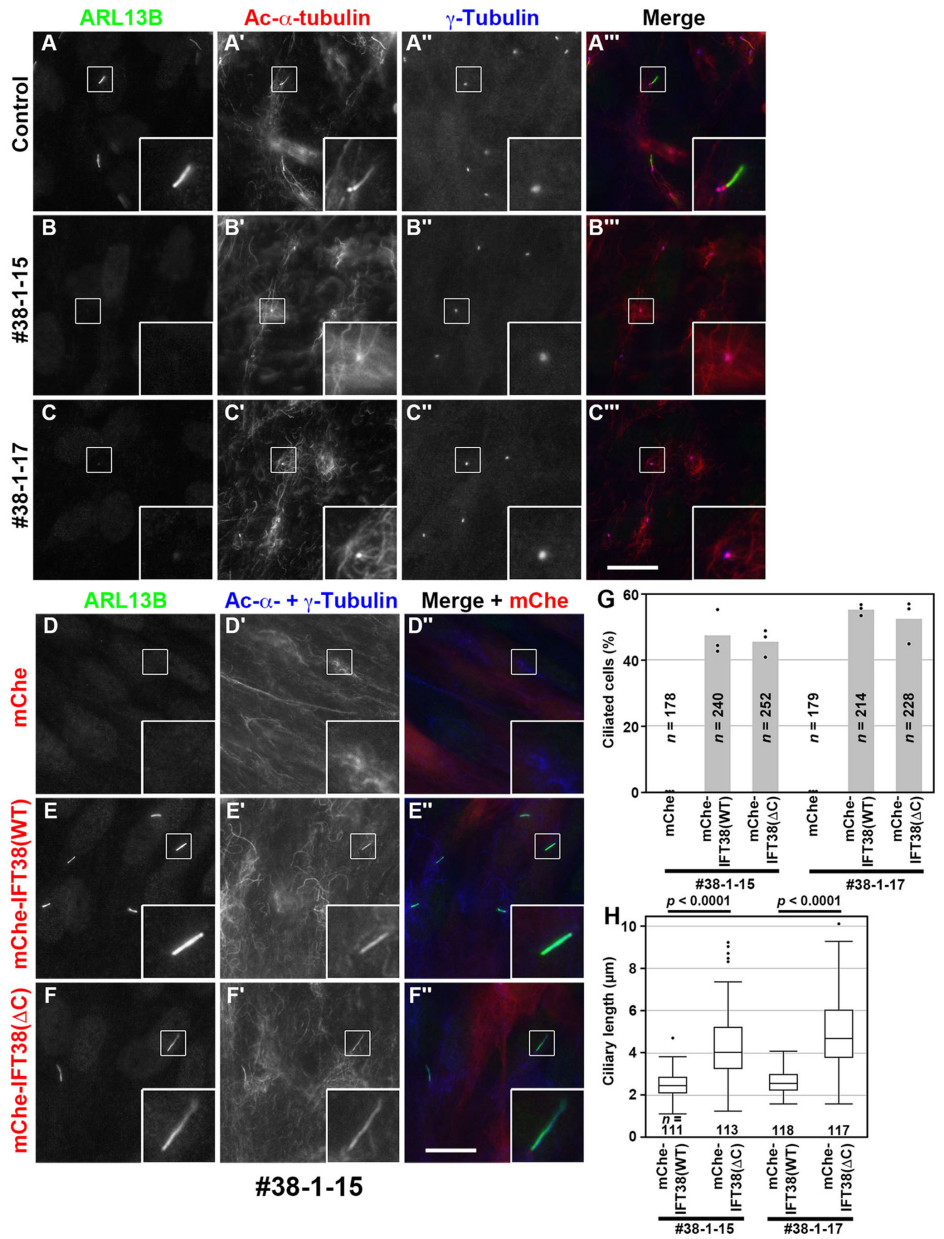
**IFT38(WT) and IFT38(ΔC) differentially** **rescue ciliogenesis** **defects of *IFT38-*KO cells.** (A–C) Control RPE1 cells and the *IFT38*-KO cell lines #38-1-15 and #38-1-17 were serum starved for 24 h and triple immunostained for IFT88 (A–C), acetylated (Ac)-α-tubulin (A′–C′), and γ-tubulin (A″–C″). (A‴–C‴) Merged images. (D–F) The *IFT38*-KO cell line #38-1-15, which stably expresses mChe (D), mChe-IFT38(WT) (E), or mChe-IFT38(ΔC) (F) were immunostained for ARL13B (D–F) and Ac-α-tubulin+γ-tubulin (D′–F′). (D″–F″) Merged images. Scale bars: 10 µm. Insets are threefold enlarged images of the boxed regions. (G) Ciliated cells of the *IFT38*-KO cell lines #38-1-15 and #38-1-17, which stably express mChe, mChe-IFT38(WT), or mChe-IFT38(ΔC) were counted, and percentages of ciliated cells are represented as bar graphs. The data are shown as means of three independent experiments; dots indicate the percentages of ciliated cells in individual experiments. In each set of experiments, 51–108 cells were analyzed, and the total numbers of cells analyzed (*n*) are shown. (H) The length of cilia in the *IFT38*-KO cell lines #38-1-15 and #38-1-17, which stably express mChe-IFT38(WT) or mChe-IFT38(ΔC) was measured and expressed as box-and-whisker plots. The box represents the 25–75th percentiles [interquartile range (IQR)], and the median is indicated. The whiskers show the minimum and maximum within 1.5×IQR from the 25th and 75th percentiles, respectively. Outliers are indicated with dots. The total numbers of cells analyzed (*n*) are shown. *P*-values were determined by the Student’s *t*-test.

In our previous study (Katoh et al., 2016), we used mouse embryonic fibroblasts (MEFs) derived from *Ift38*-KO mice (Botilde et al., 2013) to study the roles of IFT38 in the IFT-B complex. In the present study, using the CRISPR/Cas9 system (see the Materials and Methods), we established *IFT38*-KO lines of human telomerase reverse transcriptase–immortalized retinal pigment epithelial 1 (hTERT-RPE1) cells, which have been used in a number of studies on ciliary assembly, trafficking, and functions. The phenotype of the *IFT38*-KO cells was compared with those of BBS1-KO cells (Nozaki et al., 2018) and KO cells of other IFT components, which we previously established (for review, see Nakayama and Katoh, 2018). For the following experiments, we selected two independent *IFT38*-KO cell lines, #38-1-15 and #38-1-17, both of which have a one nucleotide deletion (but at distinct nucleotides, c.51delA and c.50delG, respectively) in one IFT38 allele, and a reverse integration of the donor knock-in vector in the other allele (for detailed characterization, see Fig. S2A–C). As previously reported for *Ift38*-KO MEFs (Botilde et al., 2013; Katoh et al., 2016) and recently reported for *IFT38*-KO cell lines established from hTERT-RPE1 cells (Beyer et al., 2018), the *IFT38*-KO RPE1 cell lines we established here completely lacked cilia (Fig. 3, compare B,C with A).

To exclude the potential off-target effects of the CRISPR/Cas9 system, we performed a rescue experiment. When mChe-fused IFT38(WT), but not mChe, was stably expressed in the *IFT38*-KO cell lines #38-1-15 and #38-1-17, ciliogenesis was restored (Fig. 3D,E,G), confirming that the cilia-lacking phenotype was specific to disruption of the *IFT38* gene. On the other hand, exogenously expressed mChe-IFT38(ΔC) also restored ciliogenesis essentially to the same extent as that of mChe-fused IFT38(WT) (Fig. 3F,G), in good agreement with our previous study showing that exogenously expressed IFT38(ΔC) was able to rescue ciliogenesis defects of *Ift38*-KO MEFs (Katoh et al., 2016). These results indicate that the IFT38 CT region, which is dispensable for assembly of the IFT-B complex (see Fig. 2F), is not essential for the biogenesis of cilia. However, we noticed that *IFT38*-KO cells expressing IFT38(ΔC) tended to grow longer cilia than IFT38(WT)-expressing cells (Fig. 3, compare E,F; also see Fig. S3B,C). Indeed, the difference in ciliary length between the IFT38(WT)-expressing and IFT38(ΔC)-expressing cells was statistically significant for both #38-1-15 and #38-1-17 cell lines (Fig. 3H; the median values, 2.45 versus 4.02 µm and 2.56 versus 4.68 µm, respectively; also see Fig. S3E). Although we do not know the exact reason for the elongated cilia in IFT38(ΔC)-expressing cells, this might be implicated in the increased levels of certain proteins within cilia; for example, we have recently shown that disruption of the dynein-2 intermediate chains results not only in the increased levels of some ciliary proteins but also in increased ciliary length (Tsurumi et al., 2019). On the other hand, *IFT38*-KO cells exogenously expressing mChe-IFT38(ΔN), which lacks the ability to bind IFT80 (see Fig. 2D) but retains the ability to bind the BBSome subunits (see Fig. 2B,C), had very short or vestigial cilia (Fig. S3D,E). Although, in the present study, we did not pursue the molecular basis for the very short cilia-phenotype of the IFT38(ΔN)-expressing *IFT38*-KO cells, the IFT38-IFT80 interaction might be implicated in biogenesis of cilia.

### Ciliary localization of the IFT machinery or the BBSome is not significantly altered in IFT38(ΔC)-expressing *IFT38-*KO cells

**Fig. 4.**
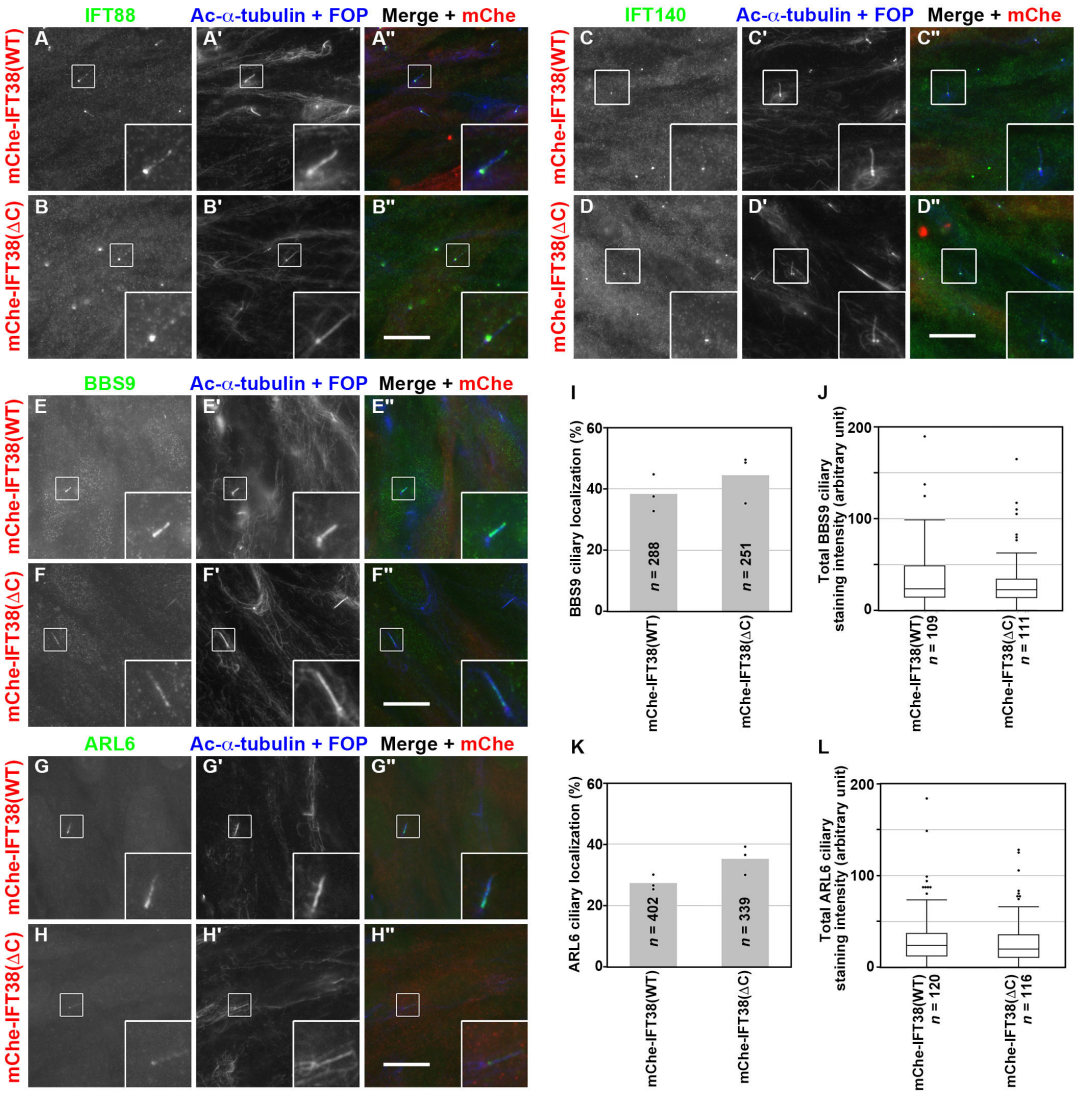
**Ciliary localization of the IFT machinery or the** **BBSome** **is not significantly altered in IFT38(ΔC)-expressing *IFT38-*KO cells.** (A–D) The *IFT38*-KO cell line #38-1-15, which stably expresses mChe-IFT38(WT) or mChe-IFT38(ΔC), was serum starved for 24 h, and immunostained for IFT88 (A,B), IFT140 (C,D), BBS9 (E,F), or ARL6 (G,H), together with Ac-α-tubulin+FOP (A′–H′). (A″–H″) Merged images. Scale bars: 10 µm. Insets are threefold enlarged images of the boxed regions. (I,K) mChe-IFT38(WT)-expressing or mChe-IFT38(ΔC)-expressing *IFT38*-KO cells with ciliary localization of BBS9 (I) or ARL6 (K) were counted, and the percentages of ciliated cells with BBS9-positive (I) or ARL6-positive cilia (K) are represented as bar graphs. Values are means of three independent experiments; dots indicate the percentages of ciliated cells in individual experiments. In each experiment, 68–107 (I) and 107–151 ciliated cells (K) were analyzed, and the total numbers of ciliated cells analyzed (*n*) are shown. (J,L) Ciliary fluorescence staining intensities of BBS9 and ARL6, and ciliary length in ciliated mChe-IFT38(WT)-expressing or mChe-IFT38(ΔC)-expressing *IFT38*-KO cells were measured, and the total staining intensities of BBS9 (J) and ARL6 (L) are expressed as box-and-whisker plots as in Fig. 3H. The total numbers of analyzed cells (*n*) are shown. *P*-values were determined by the Student’s *t*-test.

We next analyzed the localization of IFT88 (an IFT-B subunit) and IFT140 (an IFT-A subunit) in IFT38(WT)-expressing and IFT38(ΔC)-expressing *IFT38*-KO cells. As shown in Fig. 4A and B, IFT88 staining was observed mainly around the base of cilia and faintly along the axoneme, as in control RPE1 cells (for example, see Nozaki et al., 2018, 2017). IFT140 staining was found predominantly at the ciliary base in both IFT38(WT)-expressing and IFT38(ΔC)-expressing *IFT38*-KO cells (Fig. 4C,D), as in control RPE1 cells (Nozaki et al., 2018, 2017). Thus, the C-terminal truncation of IFT38 did not affect the localization of components of the IFT machinery.

We next analyzed the localization of the BBSome in *IFT38*-KO cells expressing mChe-fused IFT38(WT) and IFT38(ΔC). When cells were immunostained for BBS9, this protein was found within the cilia of approximately 40% of IFT38(WT)-expressing and IFT38(ΔC)-expressing *IFT38*-KO cells (Fig. 4E,F,I). Quantitative analysis demonstrated that the total ciliary staining intensity for BBS9 was not significantly different between IFT38(WT)-expressing and IFT38(ΔC)-expressing *IFT38*-KO cells (Fig. 4J), although cilia of IFT38(ΔC)-expressing cells were longer than those of IFT38(WT)-expressing cells (Fig. 3H). Essentially the same results were obtained when IFT38(WT)-expressing and IFT38(ΔC)-expressing *IFT38*-KO cells were immunostained for ARL6 (Fig. 4G,H,K,L).

### GPR161 export from cilia is impaired in IFT38-KO cells expressing IFT38(ΔC)

**Fig. 5.**
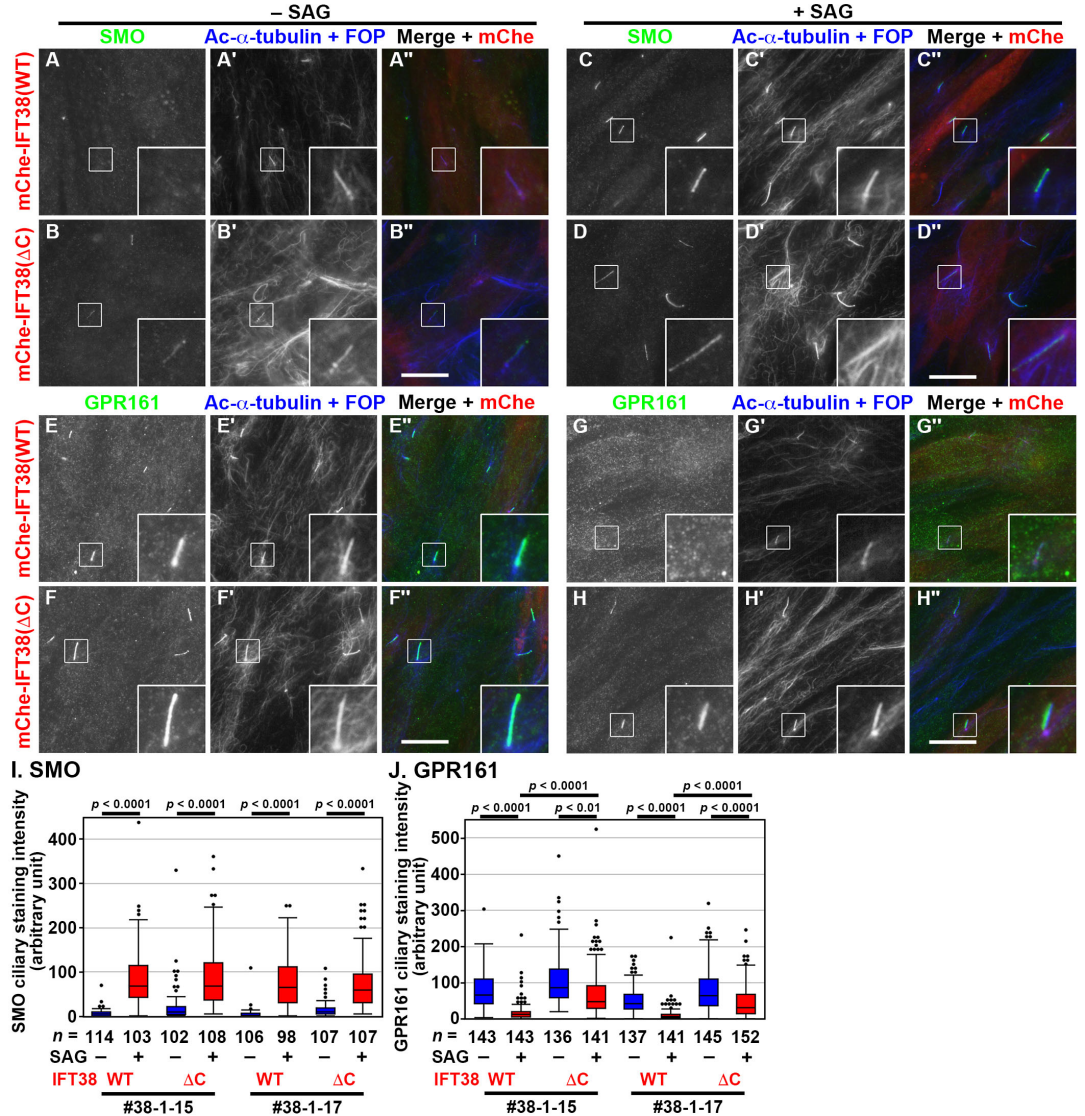
**IFT38(ΔC)-expressing *IFT38-*KO cells show** **impaired export of GPR161 upon SAG treatment.** (A–H) The *IFT38*-KO cell line #38-1-15, which stably expresses mChe-IFT38(WT) or mChe-IFT38(ΔC), was serum-starved for 24 h and further cultured for 24 h in the absence (−SAG) or presence (+SAG) of 200 nM SAG. The cells were immunostained for either SMO (A–D) or GPR161 (E–H) and Ac-α-tubulin+FOP (A′–H′). (A″–H″) Merged images. Scale bars: 10 µm. Insets are threefold enlarged images of the boxed regions. (I,J) Fluorescence staining intensities of SMO (I) and GPR161 (J) in the *IFT38*-KO cell lines #38-1-15 and #38-1-17, which stably express mChe-IFT38(WT) or mChe-IFT38(ΔC) were measured, and relative intensities of the cells are expressed as box-and-whisker plots as in Fig. 3H. *P*-values were determined by one-way ANOVA followed by Tukey post-hoc analysis for comparison among cell lines, and by the Student’s *t*-test for comparison between cells with and without SAG treatment.

As the BBSome has been shown to participate in the export of GPCRs, including GPR161, from cilia (Eguether et al., 2014; Liew et al., 2014; Nozaki et al., 2018; Ye et al., 2018), we then analyzed changes in the localization of SMO and GPR161 upon the stimulation of Hh signaling. As described previously (Nozaki et al., 2018), SMO is excluded from cilia under basal conditions but enters cilia upon treatment of cells with the small molecule activator, Smoothened Agonist (SAG), whereas GPR161 negatively regulates Hh signaling on the ciliary membrane under basal conditions but exits cilia upon SAG treatment (for review, see Mukhopadhyay and Rohatgi, 2014). In the *IFT38*-KO cell line #38-1-15, which stably expresses mChe-IFT38(WT), SMO was absent from cilia under basal conditions (Fig. 5A), whereas it was observed within cilia upon SAG treatment (Fig. 5C,I). In contrast to SMO, GPR161 was found within cilia in the absence of SAG (Fig. 5E), whereas it was exported from cilia by SAG treatment (Fig. 5G,J). Essentially the same results were obtained using the other KO cell line, #38-1-17, stably expressing IFT38(WT) (Fig. 5I,J).

We also analyzed the localization of SMO and GPR161 in *IFT38*-KO cells stably expressing mChe-IFT38(ΔC). In the absence of SAG, IFT38(ΔC)-expressing *IFT38*-KO cells tended to have slightly higher levels of SMO and GPR161 within cilia than IFT38(WT)-expressing cells, although the results were not statistically significant (Fig. 5, compare B with A, and compare F with E; also see Fig. 5I,J). Upon SAG treatment, SMO entered cilia at levels comparable between IFT38(WT)-expressing and IFT38(ΔC)-expressing *IFT38*-KO cells (Fig. 5C,D,I). On the other hand, the export of GPR161 from cilia upon stimulation with SAG in IFT38(ΔC)-expressing *IFT38*-KO cells was considerably impaired as compared with that in IFT38(WT)-expressing cells (Fig. 5, compare H with G; also see Fig. 5J).

These observations altogether indicate that the export of GPR161 from cilia is impaired in *IFT38*-KO cells expressing IFT38(ΔC). It is noteworthy that the phenotype of IFT38(ΔC)-expressing *IFT38*-KO cells, with respect to the localization of GPR161 under SAG-stimulated, but not basal, conditions, resembled that of *BBS1*-KO RPE1 cells (Nozaki et al., 2018).

## DISCUSSION

In this study, we determined, for the first time to our knowledge, the mode of direct interaction between the IFT machinery and the BBSome. Our analyses utilizing the VIP assay showed that IFT38 and three BBSome subunits, BBS1, BBS2 and BBS9 constitute the IFT-B–BBSome interface, and that the C-terminal region of IFT38 is essential for its interaction with the BBSome subunits (Figs 1,2). These results are compatible with a previous interactome study indicating that IFT38/CLUAP1 and other IFT-B subunits interact, directly or indirectly, with BBSome subunits, including BBS1 and BBS9 (Boldt et al., 2016), and with a recent study, reported while this study was in progress, showing that BBS2 was co-immunoprecipitated with IFT38 (Beyer et al., 2018).

In addition to its interaction with the BBSome, our previous studies showed that IFT38 serves as a hub subunit of the IFT-B complex, as follows: (1) by directly interacting with IFT20 and IFT80, IFT38 constitutes the IFT-B peripheral (IFT-B2) subcomplex (Katoh et al., 2016); (2) composite interactions involving IFT38 and IFT57 from the peripheral subcomplex and IFT52 and IFT88 from the core (IFT-B1) subcomplex constitute the interface between the two subcomplexes (Katoh et al., 2016); and (3) the connecting tetramer, IFT38/IFT52/IFT57/IFT88, is a binding site for heterotrimeric kinesin-II (Funabashi et al., 2018). The crucial role of IFT38 in the IFT machinery is corroborated by the fact that *IFT38-KO* cells completely lack cilia (Fig. 3; also see Beyer et al., 2018; Botilde et al., 2013; Katoh et al., 2016).

On the other hand, BBS1, BBS2 and BBS9 constitute the BBSome core subcomplex (Jin et al., 2010; Katoh et al., 2015; Nachury et al., 2007) and interact with ARL6 (Jin et al., 2010; Mourão et al., 2014; Nozaki et al., 2018). Furthermore, BBS9 interacts with BBS5, which mediates the membrane association of the BBSome (Nachury et al., 2007), and with BBS8 of the BBSome linker subcomplex, which mediates association of the BBSome with pericentriolar proteins (Katoh et al., 2015).

When the IFT38(ΔC) construct, which retains the ability to interact with other IFT-B subunits but lacks the ability to interact with the BBSome subunits (Fig. 2), was expressed in *IFT38*-KO RPE1 cells, it restored the ciliogenesis defect of the KO cells, like IFT38(WT), although IFT38(ΔC)-expressing *IFT38*-KO cells grew significantly longer cilia than IFT38(WT)-expressing cells (Fig. 3). The notable defect observed in IFT38(ΔC)-expressing *IFT38*-KO cells was that export of GPR161 from cilia in response to Hh signaling stimulation was severely impaired (Fig. 5). These results together indicate that the BBSome regulates the GPR161 export in an IFT-B-dependent manner, although we cannot entirely exclude the possibility that IFT38 is directly involved in the GPR161 trafficking through its C-terminal region.

At the beginning of this study, we hypothesized three possibilities, although not mutually exclusive, regarding the role of the IFT-B–BBSome interaction in ciliary protein trafficking. The first and most intuitive possibility is that the IFT-B–BBSome interaction is required for ciliary entry and/or anterograde trafficking of the BBSome, as the BBSome was suggested to move along the axonemal microtubules in association with IFT particles in *Chlamydomonas* flagella and in mammalian olfactory cilia (Lechtreck et al., 2009; Williams et al., 2014). However, this possibility is unlikely, although not completely excluded, as the total amount of the BBSome within cilia was not significantly different between IFT38(WT)-expressing and IFT38(ΔC)-expressing *IFT38*-KO cells.

The second possibility is that the IFT-B–BBSome interaction is required for normal assembly/trafficking of the IFT machinery, as previous studies in *Caenorhabditis elegans* implicated the role of the BBSome in the assembly of IFT particles at the basal body (Blacque et al., 2004; Wei et al., 2012), although our previous study indicated that assembly and trafficking of the IFT machinery appeared normal in *BBS1*-KO RPE1 cells (Nozaki et al., 2018). However, this possibility is also unlikely because the localization of an IFT-B (IFT88) or an IFT-A (IFT140) subunit was not different between IFT38(WT)-expressing and IFT38(ΔC)-expressing *IFT38*-KO cells.

The third possibility is that the BBSome mediates retrograde trafficking and/or export of some GPCRs across the transition zone in a manner dependent on its interaction with the IFT machinery, in view of direct binding of the BBSome to GPR161 indicated by *in vitro* experiments (Klink et al., 2017; Ye et al., 2018) and recent observations of us and others for roles of the BBSome (Liu and Lechtreck, 2018; Nozaki et al., 2018; Ye et al., 2018). Indeed, the exit of GPR161 from cilia upon stimulation of cells with SAG was significantly impaired in IFT38(ΔC)-expressing *IFT38*-KO cells. In support of this possibility, previous single molecule-imaging analyses of ciliary membrane proteins indicate that these proteins move on the ciliary membrane predominantly by diffusion and associate with the IFT machinery in a transient and stochastic manner (Milenkovic et al., 2015; Ye et al., 2013, 2018). Furthermore, a recent study on the BBSome architecture based on single-particle cryo-electron microscopy (Chou et al., 2019), in conjunction with a previous single molecule-imaging study (Ye et al., 2018), suggests that the BBSome exists in an auto-inhibited state in the absence of bound ARL6 and its conformational change induced by ARL6 binding triggers membrane binding of the BBSome and increases its interaction with cargo membrane proteins to promote their crossing of the transition zone. Taken into account the data presented in this study, it is also possible that binding of IFT-B induces a conformational change of the BBSome to allow subsequent ARL6 binding.

Involvement of the IFT-B complex in the export of ciliary GPR161 was somewhat unexpected, as it has long been believed that the IFT-B and IFT-A complexes mediate anterograde and retrograde protein trafficking driven by the kinesin-2 and dynein-2 motors, respectively (for example, see Ishikawa and Marshall, 2011). Indeed, we recently identified an interaction interface between the IFT-B complex and the anterograde kinesin-2 motor, and showed that this interaction is essential for ciliogenesis (Funabashi et al., 2018). On the other hand, however, we and others have shown that, in addition to its role in retrograde trafficking, the IFT-A complex, as well as its adaptor protein TULP3, mediates the import of ciliary GPCRs (Badgandi et al., 2017; Hirano et al., 2017; Mukhopadhyay et al., 2010; Park et al., 2013). It thus seems likely that, in addition to their roles in intraciliary trafficking, the IFT-A and IFT-B complexes participate in import and export across the ciliary gate of at least some of ciliary GPCRs via their adaptors, TULP3 and the BBSome, respectively.

The phenotype with respect to the ciliary GPR161 level in IFT38(ΔC)-expressing *IFT38*-KO cells resembles, although less striking, that observed in *BBS1*-KO and *ARL6*-KO cells (Liew et al., 2014; Nozaki et al., 2018). On the other hand, the basal or SAG-stimulated level of SMO within cilia in IFT38(ΔC)-expressing *IFT38*-KO cells is not significantly different from that in IFT38(WT)-expressing *IFT38*-KO cells. Thus, the phenotype with respect to the ciliary SMO level was different from that observed in *BBS1*-KO cells (Nozaki et al., 2018). Although we do not know the exact reason for the apparent discrepancy, it might be attributable to the difference in the BBSome integrity; namely, it is intact in IFT38(ΔC)-expressing *IFT38*-KO cells, whereas it is disrupted in *BBS1*-KO cells. Thus, the intact BBSome in IFT38(ΔC)-expressing *IFT38*-KO cells might somehow mediate the exit of SMO. On the other hand, a previous single molecule-imaging study showed that, in unstimulated cells, SMO is rarely detectable on the ciliary membrane probably due to a low level of constitutive cycling between the ciliary and plasma membranes, but occasionally undergoes sub-second confinements to a structure at the ciliary base (Milenkovic et al., 2015); the structure might represent the intermediate compartment between the transition zone and transition fiber proposed by another single molecule study (Ye et al., 2018). Therefore, in order to determine whether the IFT-B–BBSome interaction participates in the infrequent SMO crossing of the transition zone, single molecule analysis will be required in the future study.

The phenotype of IFT38(ΔC)-expressing *IFT38*-KO cells is also different from that of cells derived from *Ift25*-KO and *Ift27*-KO mice, in which the ciliary levels of the BBSome, GPR161 and SMO are significantly increased as compared to control cells (Eguether et al., 2014; Keady et al., 2012; Liew et al., 2014; Mick et al., 2015). In contrast to the direct interaction of IFT38 with the BBSome shown in this study, two previous studies independently proposed, although through distinct mechanisms, that IFT27 and its binding partner IFT25 indirectly regulate the assembly and/or function of the BBSome, and thereby regulate retrograde trafficking or export of ciliary GPCR (Eguether et al., 2014; Liew et al., 2014); in support of this, we could not detect a direct interaction of IFT25–IFT27 with the BBSome (Fig. S1). Thus, it is apparent that IFT38 and IFT25–IFT27 participate in the regulation of the BBSome function and thereby GPCR trafficking by distinct mechanisms.

Retrograde trafficking, and probably export, of ciliary proteins are mediated by the IFT-A complex with the aid of the dynein-2 motor. In our present study, however, we were unable to confirm a direct interaction of the BBSome with the IFT-A complex, although we could not completely exclude the potential IFT-A–BBSome interaction. On the other hand, we did observe the IFT-B–BBSome interaction (Fig. 1), which is supported by previous interactome studies (Beyer et al., 2018; Boldt et al., 2016). Given that the BBSome mediates the lateral transport of ciliary GPCR across the transition zone (Ye et al., 2018), the most likely mechanism for export of ciliary GPR161 is as follows. First, the BBSome connects ciliary GPR161 to the IFT-B complex. Second, the IFT-B and IFT-A complexes assemble into the IFT machinery. Third, lateral transport across the ciliary gate is powered by the dynein-2 motor, which is associated with the IFT-A complex. In any case, understanding the full picture of the roles of the very large IFT machinery, composed of 22 subunits (16 from IFT-B and six from IFT-A), and the BBSome in ciliary protein trafficking will require elucidation of the intricate roles of individual subunits in the context of protein–protein interactions.

## MATERIALS AND METHODS

### Plasmids

Expression vectors for IFT-B and BBSome subunits, and their deletion mutants constructed in this study are listed in Table S1. Construction of the other expression vectors for subunits of the IFT-A, IFT-B, and BBSome complexes were described previously (Hirano et al., 2017; Katoh et al., 2015, 2016; Nozaki et al., 2018).

### Antibodies and reagents

The antibodies used in this study are listed in Table S2. GST-tagged anti-GFP Nb pre-bound to glutathione–Sepharose 4B beads were prepared as described previously (Katoh et al., 2018, 2015). Polyethylenimine Max and SAG were purchased from Polysciences and Enzo Life Sciences, respectively.

### VIP assay and immunoblotting analysis

The VIP assay and subsequent immunoblotting analysis were carried out as described previously (Katoh et al., 2015, 2016) with slight modifications, as follows: HEK293T cells expressing EGFP-tagged and tRFP-tagged proteins were lysed in HMDEKN cell lysis buffer (10 mM HEPES, pH 7.4, 5 mM MgSO_4_, 1 mM DTT, 0.5 mM EDTA, 25 mM KCl, 0.05% NP-40) (Nishijima et al., 2017). Experimental details of the VIP assay have been recently described (Katoh et al., 2018).

### Establishment of IFT38-KO cell lines using the CRISPR/Cas9 system

The strategy for disruption of genes in hTERT-RPE1 cells (American Type Culture Collection, CRL-4000) by the CRISPR/Cas9 system using homology-independent DNA repair was previously described in detail (Katoh et al., 2017) (also see Funabashi et al., 2018; Hamada et al., 2018; Nozaki et al., 2018; Takahara et al., 2018; Takei et al., 2018). Briefly, single guide RNA (sgRNA) sequences targeting the human *IFT38/CLUAP1* gene (see Table S3) were designed using CRISPR design (Hsu et al., 2013). Double-stranded oligonucleotides for the target sequences were inserted into an all-in-one sgRNA expression vector, peSpCAS9(1.1)-2× sgRNA (Addgene 80768). hTERT-RPE1 cells were grown on a 12-well plate to approximately 3.0×10^5^ cells, and transfected with the sgRNA vector (1 µg) and the donor knock-in vector, pDonor-tBFP-NLS-Neo(universal) (0.25 µg; Addgene 80767), using X-tremeGENE9 Reagent (Roche Applied Science). After culturing in the presence of G418 (600 µg/ml), cells with nuclear tBFP signals were isolated. Genomic DNA was extracted from the isolated cells, and subjected to PCR using KOD FX Neo DNA polymerase (Toyobo), and three sets of primers (Table S3) to distinguish the following three states of integration of the donor knock-in vector: forward integration (Fig. S2A,b), reverse integration (Fig. S2A,c), and no integration with a small indel (Fig. S2A,a) (see Katoh et al., 2017). Direct sequencing of the genomic PCR products was performed to confirm the disruption of both alleles of the *IFT38* gene.

### Preparation of lentiviral vectors and cells stably expressing mChe-tagged IFT38 constructs

Lentiviral vectors for the stable expression of IFT38 constructs were prepared in a previously described manner (Takahashi et al., 2012). Briefly, pRRLsinPPT-mChe-IFT38(WT) or pRRLsinPPT-mChe -IFT38(ΔC) was transfected into HEK293T cells using Polyethylenimine Max together with the packaging plasmids (pRSV-REV, pMD2.g, and pMDL/pRRE; kind gifts from Peter McPherson, McGill University, Canada) (Thomas et al., 2009). Culture medium was replaced 8 h after transfection, and collected at 24, 36, and 48 h after transfection. The viral particle-containing medium was passed through a 0.45-µm filter and centrifuged at 32,000× ***g*** at 4°C for 4 h to precipitate viral particles, which were resuspended in Opti-MEM (Invitrogen) and stored at −80°C until use. Control RPE1 cells and *IFT38*-KO cells that stably express an mChe-IFT38 construct were prepared by the addition of the lentiviral suspension to culture medium.

### Immunofluorescence analysis

Induction of ciliogenesis and subsequent immunofluorescence analysis of hTERT-RPE1 cells were performed as described previously (Hirano et al., 2017; Nozaki et al., 2018, 2017). The immunostained cells were observed using an Axiovert 200M microscope (Carl Zeiss). For quantification analysis, all images were acquired under the same setting and imported as TIFF files using ImageJ software. A ROI was constructed by drawing a line of 3-point width along the ciliary signal of Ac-α-tubulin using a segmented line tool. To correct for local background intensity, the ROI was duplicated and dragged to a nearby region. Statistical analyses were performed using JMP Pro 13 software (SAS Institute), and *P*-values were determined by the Student's *t*-test or by one-way ANOVA followed by Tukey post-hoc analysis.

## Supplementary Material

Supplementary information
