# Supplementary material for: Requirement of IFT-B–BBSome complex interaction in export of GPR161 from cilia
Source: Biol Open. 2019 Aug 30;8(9):bio043786. doi: 10.1242/bio.043786 (PMC6777367; doi:10.1242/bio.043786)
Supplement: Supplementary information [file biolopen-8-043786-s1.pdf]

Supplementary information

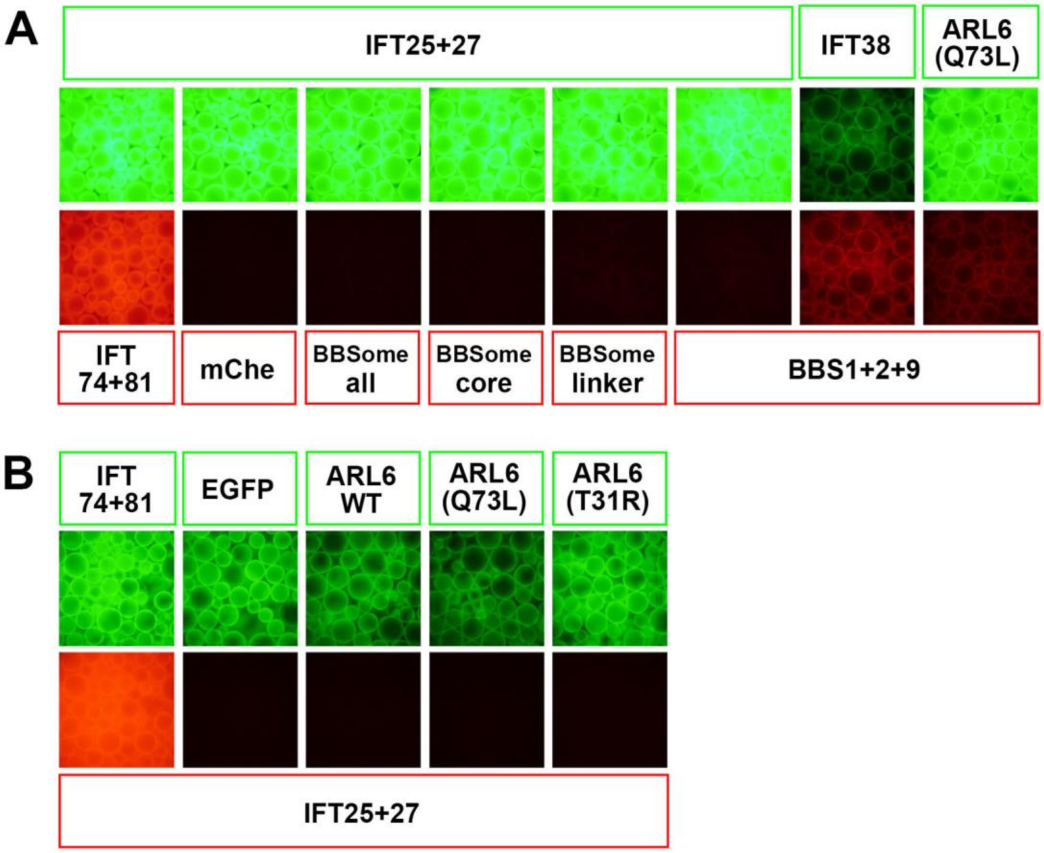

Fig. S1 Failed detection of an interaction of IFT25–IFT27 with the BBSome or ARL6 by the VIP assay

(A) Failed detection of an interaction of IFT25–IFT27 with the BBSome subunits. Lysates form HEK293T cells coexpressing EGFP-fused IFT25+IFT27, IFT38 or ARL6Δ15(Q73L) and mChe-fused BBSome subunits as indicated or IFT74+IFT81 as a positive control were subjected to the VIP assay. (B) Failed detection of an interaction of IFT25–IFT27 with ARL6. HEK293T cells coexpressing mChe-fused IFT25+IFT27 and EGFP-fused ARL6(WT), ARL6(Q73L), or ARL6(T31R) or IFT74+IFT81 as a positive control were subjected to the VIP assay.

Biology Open • Supplementary information

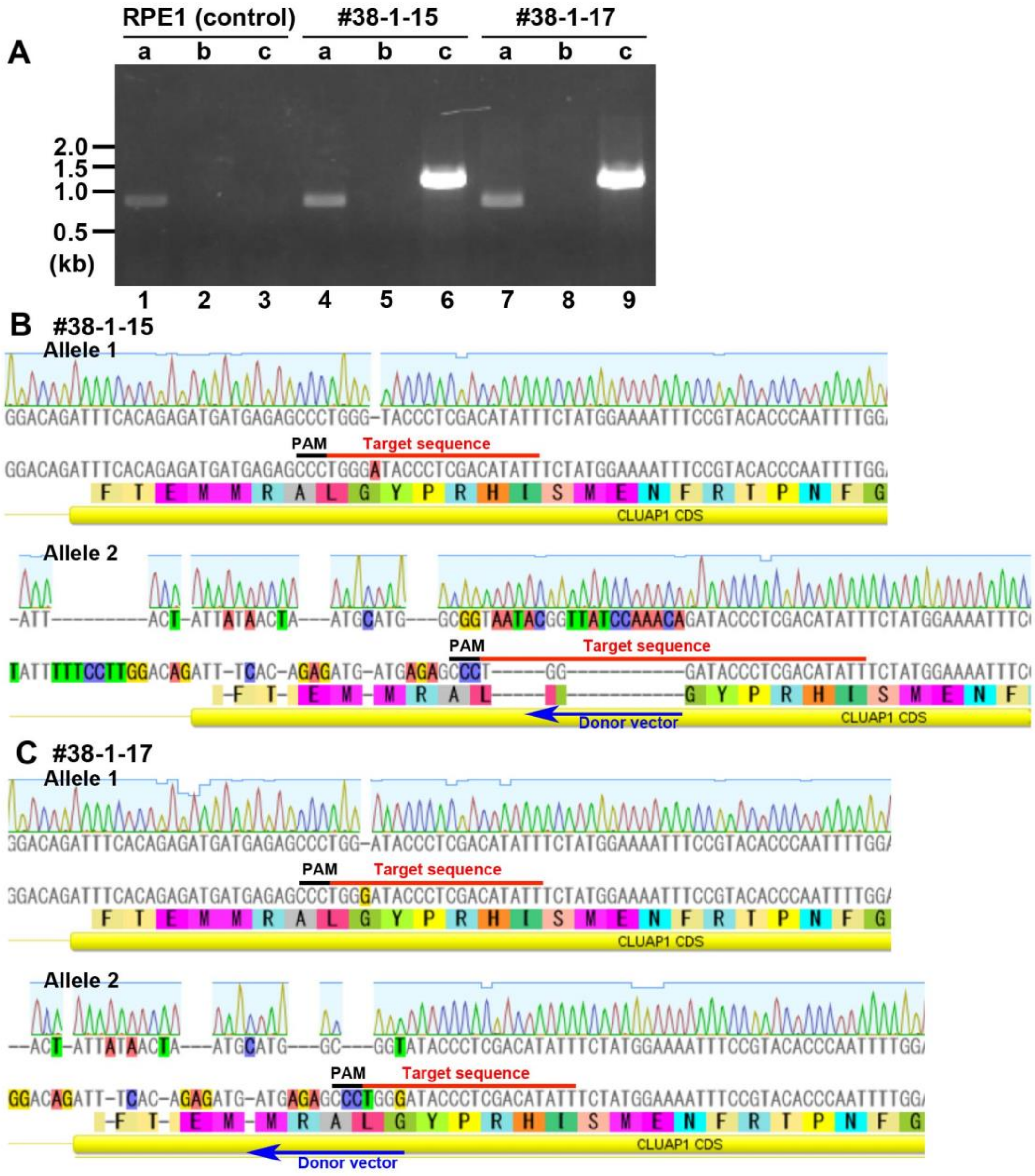

Fig. S2. Genomic PCR and sequence analyses of the *IFT38*-KO cell lines

Genomic DNAs extracted from control hTERT-RPE1 cells and from the *IFT38*-KO cell lines #38-1-15 and #38-1-17, which were established using a donor knock-in vector containing the target sequence, were subjected to PCR analysis using the indicated primer sets (see Table S2) to detect alleles with a small indel or no insertion (a), or with forward (b) or reverse (c) integration of the donor knock-in vector. (B and C) Alignments of allele sequences of the #38-1-15 (B) and #38-1-17 (C) cell lines determined by sequencing of the PCR products shown in (A). Red and black lines indicate the target sequence and PAM sequence, respectively, and blue arrows indicate the direction of integration of the donor knock-in vector.

Biology Open • Supplementary information

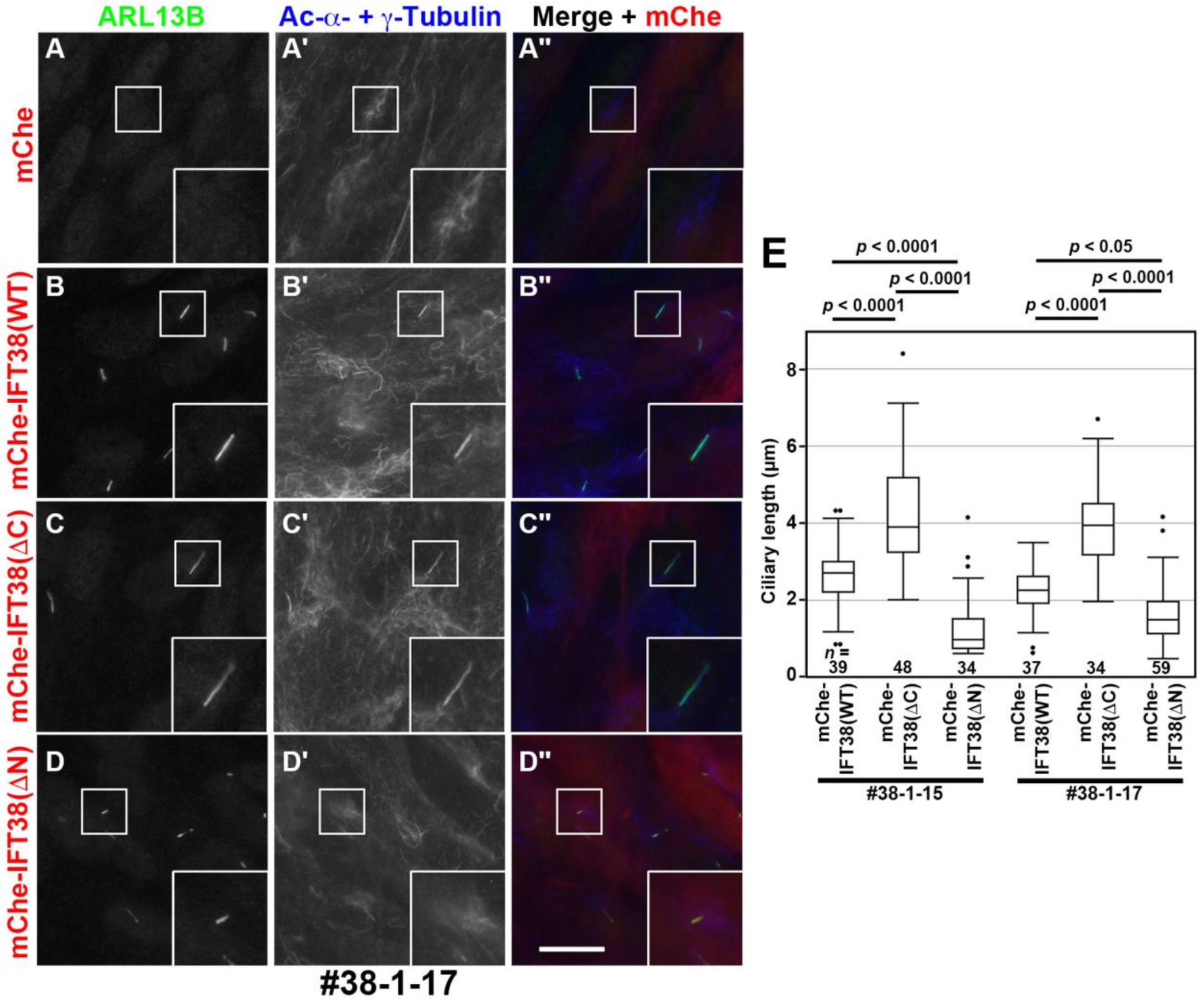

Fig. S3. Differential phenotypes of IFT38-KO cells expressing IFT38(WT), IFT38(ΔC), and IFT38(ΔN)

(A–D) The *IFT38*-KO cell line #38-1-17, which stably expresses mChe (A), mChe-IFT38(WT) (B), IFT38(ΔC) (C), or mChe-IFT38(ΔN) (D) were immunostained for ARL13B (A–D) and Ac-α-tubulin+γ-tubulin (A′–D′). Scale bar, 10 μm. (E) The length of cilia in the *IFT38*-KO cell lines #38-1-15 and #38-1-17, which stably express mChe, mChe-IFT38(WT), mChe-IFT38(ΔC), or mChe-IFT38(ΔN) was measured and expressed as box-and-whisker plots as in Fig. 3H. *p*-values were determined by one-way ANOVA followed by Tukey post-hoc analysis.

Biology Open • Supplementary information

Table S1 Plasmid vectors used in this study

Vector	Insert	Reference
pEGFP-C1	Mouse IFT38	Katoh et al. (2016)
pEGFP-C1	Mouse IFT38(ΔC: 1–328)	Katoh et al. (2016)
pEGFP-C1	Mouse IFT38(ΔN: 120–413)	Katoh et al. (2016)
pEGFP-C1	Mouse IFT38(NN-CH: 1–119)	Katoh et al. (2016)
pEGFP-C1	Mouse IFT38(CC: 120–328)	Katoh et al. (2016)
pEGFP-C1	Mouse IFT38(CT: 329–413)	Katoh et al. (2016)
pTagRFP-T-C	Human BBS1	Katoh et al. (2015)
pTagRFP-T-C	Human BBS2	Katoh et al. (2015)
pTagRFP-T-C	Human BBS4	Katoh et al. (2015)
pTagRFP-T-C	Human BBS5	Katoh et al. (2015)
pTagRFP-T-C	Human BBS7	Katoh et al. (2015)
pTagRFP-T-C	Human BBS8	Katoh et al. (2015)
pTagRFP-T-C	Human BBS9	Katoh et al. (2015)
pTagRFP-T-C	Human BBS18	Katoh et al. (2015)
pcDNA3-EGFP-N	Human ARL6	This study
pcDNA3-EGFP-N	Human ARL6(T31R)	This study
pcDNA3-EGFP-N	Human ARL6(Q73L)	This study
pEGFP-C1	Human ARL6(ΔN: 17–186,Q73L)	Nozaki et al. (2018)
pTagRFP-T-C	Human ARL6(ΔN: 17–186,Q73L)	Nozaki et al. (2018)
pCAG-mCherry-C	Human BBS2	This study
pCAG-mCherry-C	Human BBS1	This study
pCAG-mCherry-C	Human BBS9	This study
pEGFP-N1	Human IFT20	Katoh et al. (2016)
pEGFP-C1	Human IFT22	Katoh et al. (2016)
pEGFP-C1	Human IFT25	Katoh et al. (2016)
pEGFP-C1	Human IFT27	Katoh et al. (2016)
pCAG-EGFP-C	Human IFT46	Katoh et al. (2016)
pCAG-EGFP-C	Human IFT52	Katoh et al. (2016)
pEGFP-C1	Human IFT54	Katoh et al. (2016)
pCAG-EGFP-C	Human IFT56	Katoh et al. (2016)
pEGFP-C1	Human IFT57	Katoh et al. (2016)
pCAG-EGFP-C	Human IFT70B	Katoh et al. (2016)

Biology Open • Supplementary information

pCAG-EGFP-C	Human IFT74	Katoh et al. (2016)
pCAG-EGFP-C	Human IFT80	Katoh et al. (2016)
pCAG-EGFP-C	Human IFT81	Katoh et al. (2016)
pCAG-EGFP-C	Human IFT88	Katoh et al. (2016)
pCAG-EGFP-C	Human IFT172	Katoh et al. (2016)
pEGFP-C1	Human IFT43	Hirano et al. (2017)
pCAG2-EGFP-C	Human IFT121	Hirano et al. (2017)
pCAG2-EGFP-C	Human IFT122	Hirano et al. (2017)
pCAG2-EGFP-C	Human IFT139	Hirano et al. (2017)
pCAG2-EGFP-C	Human IFT140	Hirano et al. (2017)
pCAG2-EGFP-C	Human IFT144	Hirano et al. (2017)
pEGFP-C1	Human TULP3	Hirano et al. (2017)
pTagRFP-T-N	Human IFT20	Katoh et al. (2016)
pTagRFP-T-C	Human IFT25	Katoh et al. (2016)
pTagRFP-T-C	Human IFT27	Katoh et al. (2016)
pCAG-mCherry-C	Human IFT54	Katoh et al. (2016)
pCAG2-mCherry-C	Human IFT57	This study
pCAG-mCherry-C	Human IFT74	Katoh et al. (2016)
pCAG-mCherry-N	Human IFT80	Katoh et al. (2016)
pCAG-mCherry-C	Human IFT81	Katoh et al. (2016)
pCAG-mCherry-C	Human IFT88	Katoh et al. (2016)
pRRLsinPPT-mCherry-C	Mouse IFT38	This study
pRRLsinPPT-mCherry-C	Mouse IFT38(ΔC: 1–328)	This study
pRRLsinPPT-mCherry-C	Mouse IFT38(ΔN: 120–413)	This study

Biology Open • Supplementary information

Table S2 Antibodies used in this study

Antibody	Manufacturer	Clone or catalog number	Dilution (purpose)
Monoclonal mouse anti-Ac-α-tubulin	Sigma-Aldrich	6-11B-1	1:1,000 (IF)
Monoclonal mouse anti-γ-tubulin	Sigma-Aldrich	GTU-88	1:1,000 (IF)
Polyclonal rabbit anti-ARL13B	Proteintech	17711-1-AP	1:1,000 (IF)
Polyclonal rabbit anti-IFT88	Proteintech	13967-1-AP	1:1,000 (IF)
Polyclonal rabbit anti-IFT140	Proteintech	17460-1-AP *	1:500 (IF)
Polyclonal rabbit anti-ARL6	Proteintech	12676-1-AP	1:500 (IF)
Polyclonal rabbit anti-GPR161	Proteintech	13398-1-AP	1:200 (IF)
Polyclonal rabbit anti-BBS9	Atlas Antibodies	HPA021289	1:1,000 (IF)
Polyclonal rabbit anti-SMO	Abcam	ab38686	1:500 (IF)
Monoclonal mouse anti-FGFR1OP	Abnova	2B1	1:10,000 (IF)
Monoclonal mouse anti-RFP	MBL Life Science	3G5	1:1,000 (IF)
Polyclonal rabbit anti-RFP	MBL Life Science	PM005	1:1,000 (IB)
Polyclonal rabbit anti-mCherry	Proteintech	26765-1-AP	1:1,000 (IB)
Monoclonal mouse anti-GFP	BD Biosciences	JL-8	1:1,000 (IB)
AlexaFluor-conjugated secondary	Molecular Probes	A11034, A21240, A21147, A21242	1:1,000 (IF)
DyLight 649-conjugated secondary	Jackson ImmunoResearch	115-495-209	1:500 (IF)
Peroxidase-conjugated secondary	Jackson ImmunoResearch	115-035-166, 111-035-144	1:3,000 (IB)

Notes:

IF, immunofluorescence; IB, immunoblotting

*A new lot of the anti-IFT140 antibody (17460-1-AP) was used to detect the IFT140 protein around the ciliary base, without nonspecific staining in the nucleus, as observed in our previous studies.

Biology Open • Supplementary information

Table S3 Oligodeoxyribonucleotides used in this study

Name	Sequence
IFT38-gRNA#1-S	5'-AATATGTCGAGGGTATCCCA-3'
IFT38-gRNA#1-AS	5'-TGGGATACCCTCGACATATT-3'
IFT38-gRNA#2-S	5'-TCAGGCGGGATGTCAGTCTG-3'
IFT38-gRNA#2-AS	5'-CAGACTGACATCCCGCCTGA-3'
IFT38-Genome-#1-S	5'-TGAATGTCATCCTACGGATTCCCC-3'
IFT38-Genome-#1-AS	5'-AACTATGAGTGTGTACCACCCAGG-3'
IFT38-Genome-#2-S	5'-CTAATCGGCCCTAGCACTGATCTC-3'
IFT38-Genome-#2-AS	5'-CCACAGAAACCCAGCAATGGAATG-3'
pTagBFP-N-RV2	5'-CGTAGAGGAAGCTAGTAGCCAGG-3'

Supplementary References

Hirano, T., Katoh, Y. and Nakayama, K. (2017). Intraflagellar transport-A complex mediates ciliary entry and retrograde trafficking of ciliary G protein-coupled receptors. *Mol. Biol. Cell* **28**, 429-439.

Katoh, Y., Nozaki, S., Hartanto, D., Miyano, R. and Nakayama, K. (2015). Architectures of multisubunit complexes revealed by a visible immunoprecipitation assay using fluorescent fusion proteins. *J. Cell Sci.* **128**, 2351-2362.

Katoh, Y., Terada, M., Nishijima, Y., Takei, R., Nozaki, S., Hamada, H. and Nakayama, K. (2016). Overall architecture of the intraflagellar transport (IFT)-B complex containing Cluap1/IFT38 as an essential component of the IFT-B peripheral subcomplex. *J. Biol. Chem.* **291**, 10962-10975.

Nozaki, S., Katoh, Y., Kobayashi, T. and Nakayama, K. (2018). BBS1 is involved in retrograde trafficking of ciliary GPCRs in the context of the BBSome complex. *PLoS ONE* **13**, e0195005.

Biology Open • Supplementary information
